# Ultralow temperature terahertz magnetic thermodynamics of perovskite-like SmFeO_3_ ceramic

**DOI:** 10.1038/srep14777

**Published:** 2015-10-01

**Authors:** Xiaojian Fu, Xinxi Zeng, Dongyang Wang, Hao Chi Zhang, Jiaguang Han, Tie Jun Cui

**Affiliations:** 1 State Key Laboratory of Millimeter Waves, School of Information Science and Engineering, Southeast University, Nanjing 210096, China; 2 State Key Laboratory of New Ceramics and Fine Processing, School of Materials Science and Engineering, Tsinghua University, Beijing 100084, China; 3 Center for Terahertz Waves and College of Precision Instrument and Optoelectronics Engineering, Tianjin University, Tianjin 300072, China; 4 Cooperative Innovation Centre of Terahertz Science, No.4, Section 2, North Jianshe Road, Chengdu 610054, China

## Abstract

The terahertz magnetic properties of perovskite-like SmFeO_3_ ceramic are investigated over a broad temperature range, especially at ultralow temperatures, using terahertz time-domain spectroscopy. It is shown that both resonant frequencies of quasi-ferromagnetic and quasi-antiferromagnetic modes have blue shifts with the decreasing temperature due to the enhancement of effective magnetic field. The temperature-dependent magnetic anisotropy constants are further estimated using the resonant frequencies, under the approximation of omitting the contribution of Sm^3+^ magnetic moments to the effective field. Specially, the effective anisotropy constants in the *ca* and *cb* planes at 3 K are 6.63 × 10^5^ erg/g and 8.48 × 10^5^ erg/g, respectively. This thoroughly reveals the terahertz magnetic thermodynamics of orthoferrites and will be beneficial to the application in terahertz magnetism.

Rare earth orthoferrites with distorted perovskite structure have received lots of attention in recent years^1^,^2^,^3^. This series of compounds have been found to possess G-type antiferromagnetic ordering formed by Fe^3+^ ions spins and the precession frequency of magnetic moments can extend to terahertz regime due to the strong internal magnetic field^4^,^5^. Besides, the canted spins also induce weak macroscopic magnetization and ferroelectricity in some members^6^. Therefore, ReFeO_3_-type oxides exhibit abundant physical properties such as the terahertz magnetic response, the muitiferroic and magneto-optical effect^7^,^8^,^9^,^10^,^11^,^12^.

There are usually three competitive exchange interactions in orthoferrites induced by Fe-Fe, Re-Fe, and Re-Re, respectively. The Fe-Fe interaction determines the formation of antiferromagnetic ordering in the high temperature region, while the Re-Fe exchange effect will lead to magnetic anisotropy and further induce the spin reorientation (SR)^13^. However, the Re-Re interaction will be activated at very low temperature, which contributes to the long range magnetic ordering of rare earth ions. For example, SmFeO_3_ exhibits antiferromagnetic ordering along *a* axis with a net spontaneous magnetization along *c* axis below 670 K (Neel temperature). Then, the magnetic moments continuously rotate from *a* axis to *c* axis during 450 ~ 480 K due to the Sm-Fe interaction. The formation of long range magnetic ordering in Sm sublattice plays an important role in determining the macroscopic magnetic properties at very low temperature. Magnetization reversal was observed in SmFeO_3_ below 5 K under a magnetic field about 300 ~ 500 Oe, which can be ascribed to the antiparallel ferromagnetic moments of Fe sublattice and Sm sublattice^6^,^14^. This interesting phenomenon may have potential applications in the magnetic switch under a weak applied field.

Despite the terahertz antiferromagnetic resonances and potential physical phenomena, SmFeO_3_ has not been investigated in the terahertz regime. In this work, we fabricate the SmFeO_3_ ceramic samples and characterize their terahertz magnetic properties in a wide temperature range. We will discuss the magnetic thermodynamics of the SmFeO_3_ ceramic in details, including the temperature-dependent ferromagnetic and antiferromagnetic resonant frequencies of Fe sublattice, as well as the contribution of Sm spins to macroscopic magnetization and magnetic resonance at the ultralow temperatures.

## Results and Discussion

**Figure 1:**
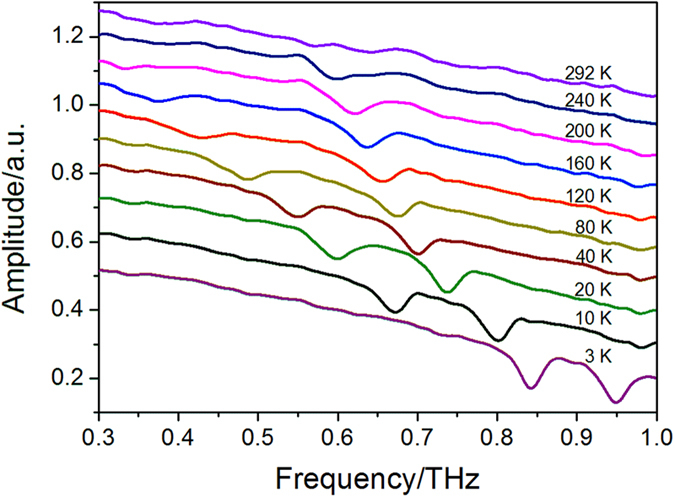
The terahertz frequency-domain transmission spectra of the SmFeO_3_ ceramic between 3 K and 292 K. The transmission amplitude is normalized to the reference spectrum. Since the transmittance at different temperatures does not remarkably differ from each other, the transmission curves are translated along y axis in order to clearly show the resonant dips.

Figure 1 shows the terahertz transmission frequency-domain spectra (normalized to the reference spectrum) of the SmFeO_3_ ceramic between 3 K and 292 K. Only partial curves are presented to keep the tendency observable. Below 200 K, two dips are observed on the transmission curves, which can be ascribed to the so-called quasi-ferromagnetic mode (F mode) and quasi-antiferromagnetic mode (AF mode) of SmFeO_3_, respectively^15^. The resonant frequencies of F mode and AF mode are 0.34 THz and 0.62 THz at 200 K, respectively. As the temperature decreases, both the resonant frequencies of two modes exhibit blue shift. At 40 K, the respective frequencies are 0.55 THz and 0.70 THz. Below 40 K, the effect of temperature on the resonant frequencies becomes much more significant. When temperature lowers to 10 K, the frequencies of two modes increase to 0.67 THz and 0.80 THz, respectively. At 3 K, F mode and AF mode further harden, whose frequencies are 0.84 THz and 0.95 THz, respectively. It is worth noting that the resonant strength weakens at high temperatures. Specially, the dip attributed to F mode cannot be resolved from the background above 200 K, while AF mode also gets very weak at room temperature (RT), with a frequency of 0.57 THz.

**Figure 2:**
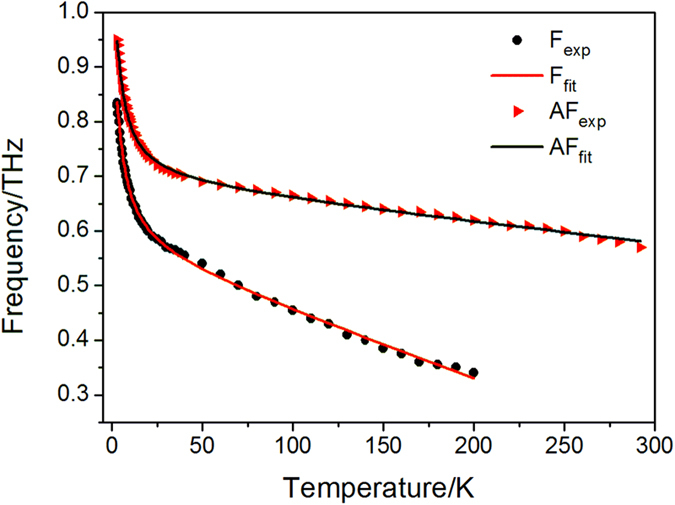
The temperature dependent resonant frequencies of F and AF modes. The solid circles and triangles represent the experimental values, whereas the solid lines are the fitting results.

The resonant frequencies of F mode and AF mode at various temperatures are extracted from the frequency-domain spectra and presented in Fig. 2. As mentioned above, the resonant frequencies for both modes undergo a sharp decrease over the range of 3 ~ 40 K, while above 40 K, the frequency-temperature curves slope gently downward, especially for the AF mode. Besides, the F mode data between 200 K and RT are not shown since it almost disappears in this temperature interval.

**Figure 3:**
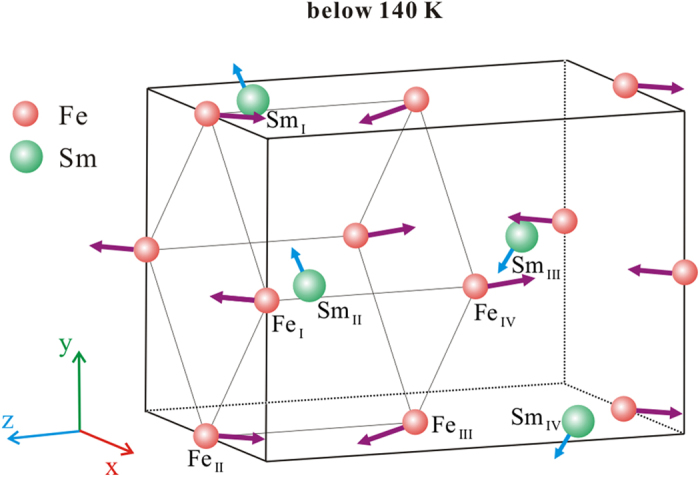
The crystal structure, atom arrangements, and spin orientations of SmFeO_3_ crystal below 140 K. The eight nearest Fe^3+^ ions constitute a cubic, whose spins orient along *c* axis with a weak macroscopic magnetization along *a* axis. By contrast, the spins of Sm^3+^ activated below 140 K locate in the ab plane and increase during cooling process.

Next, let us consider the physical origin of the resonant modes and the corresponding magnetic thermodynamics. The crystal structure of SmFeO_3_ is shown in Fig. 3. As can be seen, Fe^3+^ ions occupy the (0 0 0.5) sites, of which, there are eight edge sites and four face center sites, according to the symmetry of Pbnm space group. Besides, the eight nearest Fe^3+^ ions constitute a cubic and the spin orientations for adjacent ions are opposite, that is, G-type antiferromagnetic ordering is formed^13^. In fact, the spins of adjacent Fe^3+^ ions are not strictly antiparallel. Specifically, just below the Neel temperature (*T*_N _= 670 K), the canted spin mainly orient along *a* axis and also have a small component along *c* axis. Therefore, the magnetic structure can be denoted as 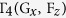, where **G** is the antiferromagnetic vector and **F** is the ferromagnetic vector. Like most other rare earth orthoferrites, SmFeO_3_ undergoes a spin reorientation transition due to the interaction between rare earth ions and Fe^3+^ ions. However, difference is that the transition temperature of SmFeO_3_ is the highest in the family of rare earth orthoferrites and much higher than RT. At about 480 K, the 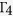 phase changes to 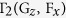 through a mesophase 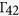
14. Thus, as seen in Fig. 3, the Fe^3+^ spins orient along *c* axis with a weak macroscopic magnetization along *a* axis below the transition temperature. The canted spins induce the weak macroscopic magnetism in SmFeO_3_ and the magnitude of magnetization depends on the ferromagnetic component of magnetic moments, while the terahertz magnetic resonances caused by the spin precession under an internal magnetic field relate to the **F** and **G** vectors.

**Figure 4:**
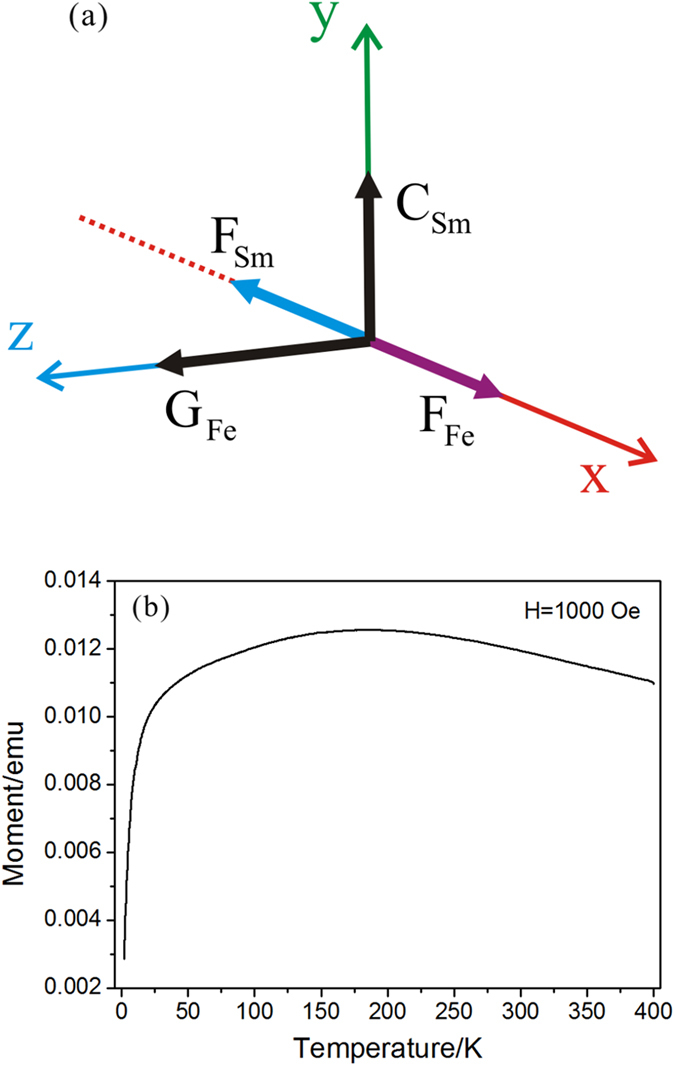
The orientation of magnetic moments and macroscopic magnetization in the SmFeO_3_ ceramic. (**a**) Magnetic vectors **F** and **G** of Sm^3+^ and Fe^3+^ ions; (**b**) the measured magnetization at various temperatures under a magnetic field of 1000 Oe.

According to some previous studies, the magnetic moments of Sm^3+^ ions play an important role on the magnetic properties of SmFeO_3_ at low temperature. Owing to the relative strong Re-Fe exchange interaction, SmFeO_3_ possess a high SR transition temperature, while the Re-Re interaction leads to a high magnetic ordering temperature for Sm^3+^ ions. At about 140 K, the Sm^3+^ ions spins are activated in the *ab* plane. As seen in Figs 3 and 4(a), Sm^3+^ ions exhibit the 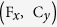 symmetry, that is, the spins satisfy the following equations: S_1x _= S_2x _= S_3x _= S_4x_ and S_1y _= S_2y _= −S_3y_ = −S_4y_. Thus, Sm^3+^ ions possess C-type antiferromagnetic ordering along *b* axis and also a ferromagnetic moment along *a* axis. Moreover, macroscopic magnetization orients along the −*a* direction, antiparallel with the one of Fe^3+^ ions^16^. During the cooling process, the remarkably increased net magnetic moment of Sm^3+^ ions will cancel with the opposite contribution from Fe^3+^ ions, which leads to a zero macroscopic magnetization at the temperature called compensation point (about 5 K). Below this temperature, magnetic reversal is observed when magnetic field is applied parallel to *a* axis in the SmFeO_3_ crystal^6^. To verify this phenomenon in the ceramic sample, we test the 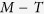 curve under an applied magnetic field of 1000 Oe. As shown in Fig. 4(b), the magnetization increases first during the cooling process due to the increased ferromagnetic component of Fe^3+^ spins, then begins to decrease gradually because of the activation of Sm^3+^ spins at about 170 K (different from the crystal sample), companied by a sharp decline below 40 K. Nevertheless, magnetic reversal does not appear in the SmFeO_3_ ceramic even when the temperature is lowered to 2 K. The possible reason is as follows. Crystal sample has a long range ordering and the macroscopic magnetization is measured when applied field is parallel to *a* axis. However, for the SmFeO_3_ ceramic, magnetic ordering is formed in a single crystal grain and the orientation of crystal grains is random, and therefore, the measured magnetization is an average value of various orientations between the magnetic field and crystal axis.

Now, let us further consider the temperature dependent magnetic resonant frequencies based on the foregoing discussions about the magnetic structure in SmFeO_3_. In antiferromagnetic materials, the resonant frequency can be described by^4^,^17^
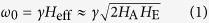
in which, 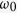 is the angular frequency, *γ* is the gyromagnetic ratio, *H*_eff_ is the effective magnetic field, while 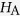 and 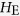 are the anisotropy field and exchange fields, respectively. For the SmFeO_3_ ceramic with 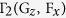 phase, the resonant frequencies of F mode and AF mode can be expressed respectively by^18^,^19^
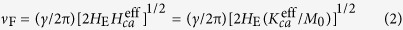

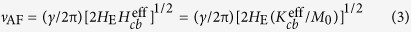
where 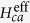 and 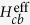, 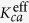 and 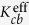 are the effective second-order anisotropy fields and anisotropy constants in the *ca* and *cb* planes respectively, and 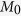 is the saturation magnetic moment. Moreover, the exchange field 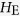 is proportional to the magnetic moment 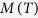, that is 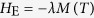, where 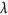 is the molecular field coefficient^4^. Since the temperature region considered in this work is much lower than the Neel temperature and no SR transition occurs, the exchange field can be regarded as nearly temperature independent^20^. However, the anisotropy field changes influenced by the Fe-Fe exchange, magnetic dipole interaction, and crystal field will change with temperature^21^. According to Eqs (2) and (3), it can be found that the square of resonant frequency is proportional to the anisotropy constant. As a consequence, we can obtain the temperature dependent anisotropy constants using the frequency data.

It is worth noting that the above discussion have not taken account of the contribution of Sm^3+^ magnetic moments to the effective field. This approximation is valid, especially for the temperature region above 40 K, as the magnetic moment of Sm^3+^ ion is much weaker than that of Fe^3+^ ion. The ground state levels for Sm^3+^ ([Xe]4f^5^) and Fe^3+^ ([Ar]3d^5^) in an octahedral crystal field are ^6^H_5/2_ and ^6^S_5/2_, respectively. According to the Hund’s rules^22^, we may conclude that the saturation magnetic moment of Fe^3+^ (5.92 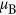, close to 5 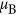 in the orthoferrites system^18^, where 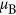 is Bohr magneton.) is large enough compared to the one of Sm^3+^ (0.85 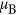, actually less than this value even at 5 K^6^), and that the effective field is mainly contributed by the magnetic moments of Fe^3+^ ions.

We fit the 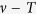 curve using the nonlinear curve fitting method. The fitting results are also presented in Fig. 2 together with the experimental data for comparison. The equations used for fitting the frequencies of F mode and AF mode can be expressed by Eq. (4) and Eq. (5), respectively.
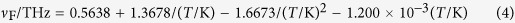

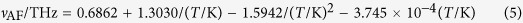


**Figure 5:**
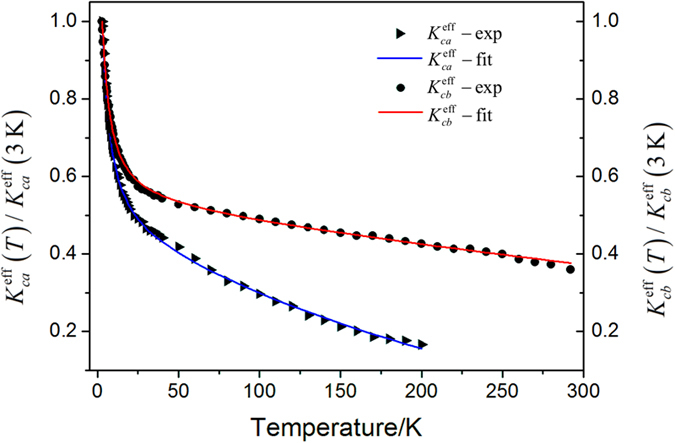
The temperature dependent magnetic effective anisotropy constants in the *ca* and *cb* planes. The solid triangles and rounds are the experimental values, and the blue and red lines represent the fitting result according to Eq. (4) and Eq. (5), respectively; the anisotropy constants are normalized to the one at 3 K.

As shown in Fig. 2, the fitting curves agree well with the experimental points for both F mode and AF mode, thus, the proposed equation is applicable in the temperature range from 3 K to RT. However, the fitting curve can be divided into three intervals due to the different tendencies. Between 40 K and RT, the resonant frequencies and temperature satisfy the linear relationship; the items 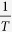 and 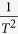 can be omitted since they are small enough compared to the linear item. The blue shift of resonant frequencies can be attributed to the increase of anisotropy constants, and hence the enhancement of effective magnetic field. It is noted that the F mode hardens fast than AF mode, which implies that growth rate of 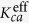 is larger than that of 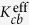. The second region is during 5 ~ 40 K. Since both the 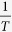 item and the linear item work in this interval, the resonant frequencies remarkably increase with decreasing temperature. The addition of 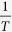 item implies that the effective anisotropy constants increase more quickly during cooling, compared to the first process. Then, below 5 K, the linear item can be deleted. However, the 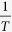 item is not enough to depict the rapidly increased frequencies, so we introduce the 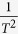 item, with which we get a good fitting (see Fig. 2). Furthermore, the effective anisotropy constants 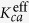 and 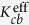
are calculated according to Eqs (2) and (3), using the resonant frequency data. The amplitudes of the anisotropy constants are normalized to the one at 3 K, and both the experimental and fitting values have been obtained and presented in Fig. 5. According to some previous studies, the exchange field in rare earth orthoferrites is about 6.4 × 10^6^ Oe^19^,^20^, and 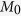 is calculated as 109.85 emu/g, and hence, the effective anisotropy constants 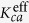 and 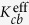
at 3 K can be estimated as 6.63 × 10^5^ erg/g and 8.48 × 10^5^ erg/g, respectively. Thus, we have obtained the temperature dependent anisotropy constants which essentially determine the magnetic resonant frequency of orthoferrites.

## Conclusions

In summary, the terahertz magnetic thermodynamics of the SmFeO_3_ ceramic have been investigated over a wide temperature region from 3 K to 292 K. The macroscopic magnetization is measured and the magnetic reversal does not occur even at 2 K for the ceramic sample. Additionally, both the F mode and AF mode of the SmFeO_3_ orthoferrite harden during the cooling process, which can be attributed to the increase of anisotropy constants, and hence the enhancement of the effective magnetic field. The resonant frequencies of both two modes can be well fitted with a nonlinear equation of temperature, which clearly describes the temperature dependence of the resonant frequencies in different temperature region. With the frequency values, we also estimate the anisotropy constants at various temperatures.

## Methods

SmFeO_3_ ceramics were fabricated by the pressureless sintering method reported in ref. 9. The phase analysis was performed on an X-ray diffractometer (Rigaku D/max2500, Japan) with CuK*α* radiation. It is shown that the ceramic belongs to the orthorhombic system and has a perovskite-like crystal structure.

**Figure 6:**
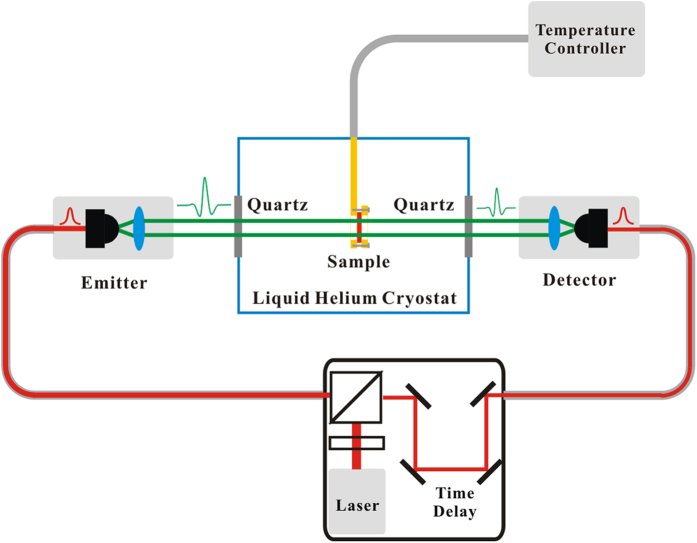
The schematic diagram of the terahertz time-domain measurement system. Terahertz pulse is excited by a 780 nm near-infrared femtosecond laser in the emitter component, and first passes through the quartz window of the liquid helium cryostat, then interacts with the sample, followed by the other quartz window, and lastly arrives at the detector component. The cooling system with liquid Helium circulation can realize the precise temperature control between 3 K and RT.

The terahertz transmission spectra were measured using a terahertz time-domain spectroscopy (TDS) system equipped with a liquid Helium cryostat. The schematic diagram of the TDS is presented is Fig. 6. As can be seen, the pump and probe near-infrared laser is transmitted in the optical fiber, and the sample to be tested is placed in a liquid Helium cryostat with precise temperature control. During the measurements, the sample was first cooled from RT to 3 K, and then, the transmitted terahertz pulses were collected between 3 K and RT during the heating process. With the time-domain data, the corresponding frequency-domain spectra can be obtained after fast Fourier transformation.

## Additional Information

**How to cite this article**: Fu, X. *et al.* Ultralow temperature terahertz magnetic thermodynamics of perovskite-like SmFeO_3_ ceramic. *Sci. Rep.* **5**, 14777; doi: 10.1038/srep14777 (2015).
